# Effects of Combined Application of Coal-Based Charcoal and Organic Fertilizer on Soil Properties and Plant Growth in Desertified Soils

**DOI:** 10.3390/plants15131963

**Published:** 2026-06-25

**Authors:** Wei Li, Xiangmeng Chen, Qing Mao, Xiaochen Yue, Wanxi Peng, Haiping Gu

**Affiliations:** 1 College of Forestry, Henan Agricultural University, Zhengzhou 450046, China; 15236900487@163.com (W.L.); yuexiaochen95@163.com (X.Y.); hpg@henau.edu.cn (H.G.); 2 College of Science, Henan Agricultural University, Zhengzhou 450046, China; xmchen0610@163.com

**Keywords:** desertification, coal-based charcoal, organic fertilizer, soil amendment, plant growth, land restoration

## Abstract

Desertified soils severely limit vegetation restoration and sustainable land use in arid regions. This study aims to evaluate the individual and combined effects of coal-based charcoal produced by coal pyrolysis and organic fertilizer on soil properties and the growth performance of *Amorpha fruticosa* L. A pot experiment was conducted using degraded sandy soil collected from Inner Mongolia, with amendment rates of 2.5%, 5%, and 10% (*w*/*w*) for each material, and a combined treatment (2.5% coal-based charcoal + 2.5% organic fertilizer). The results showed that all treatments reduced soil bulk density (BD) and increased electrical conductivity and nutrient availability. Application of coal-based charcoal increased soil pH, whereas organic fertilizer decreased it, and their combined application resulted in a more balanced soil pH. The combined treatment (FT) achieved the highest germination rate (83.33%), significantly improved root morphological traits, enhanced chlorophyll content and the photosynthetic rate, and increased peroxidase, superoxide dismutase, and catalase activities, while reducing malondialdehyde content. These findings suggest that combining coal-based charcoal with organic fertilizer provides complementary benefits, enhancing soil physicochemical properties and plant physiological performance, thereby promoting the growth of *A. fruticosa* L. and providing an effective strategy for restoring desertified soils.

## 1. Introduction

Soil formation results from the combined effects of biological factors, climate, parent material, topography, and time, leading to different soil properties [1]. Soil texture is one of the key factors affecting soil quality and productivity [2]. Sandy soils are characterized by low mineral and organic carbon (OC) content, poor fertility, few stable, large aggregates, and limited water retention capacity, all of which negatively affect plant growth [3]. China is one of the countries most severely affected by desertification. According to survey reports, by 2019, desertified land in China had reached 2.5737 million km^2^, accounting for 26.8% of the country’s total land area, with sandy desertified land covering 1.6878 million km^2^. Compared with 2014, the areas of desertified and sandy desertified land decreased by 37,880 and 33,352 km^2^, respectively [4].

For decades, improving the quality of desertified soils has been a central focus of soil and environmental research. The need to enhance soil stability and fertility arises from various practical constraints, while conventional agricultural practices continue to rely heavily on chemical fertilizers and pesticides [5]. Biochar, a carbon-rich material produced through the thermal decomposition of biomass under oxygen-limited conditions, has gained increasing attention as a soil amendment [6]. Biochar has been widely reported to enhance carbon sequestration, improve soil fertility, and reduce contaminant mobility. Owing to its unique physicochemical properties, biochar can regulate soil pH and enhance nutrient retention and water-holding capacity [7].

Furthermore, biochar’s porous structure and surface functionality enable interactions with soil microorganisms and nutrients, thereby enhancing soil functionality. In recent years, combined applications of biochar and organic amendments, such as compost, have demonstrated greater efficiency in improving soil properties than single applications. In nutrient-poor sandy soils, co-composted biochar has been shown to significantly enhance plant growth, with biomass increases of up to 305% under specific conditions [8]. Similarly, organic fertilizers play a crucial role in enhancing soil quality by supplying nutrients and mitigating the adverse effects of excessive chemical fertilizer use. Previous studies have shown that integrating organic materials, such as manure, straw, and biochar, can significantly influence soil microbial communities and nutrient cycling. For instance, manure application has been found to enhance microbial carbon and nitrogen use efficiency and promote soil carbon storage, whereas biochar increases soil organic carbon and pH and reduces the abundance of plant-parasitic organisms. Overall, incorporating organic amendments can enhance soil productivity by reshaping soil microbial and trophic structures, thereby mitigating the negative impacts of intensive chemical inputs [9].

China possesses abundant coal resources, with more than 800 million tonnes of confirmed reserves, and has established major coal-based energy production hubs in eastern Inner Mongolia [10,11]. Coal-based charcoal is produced by pyrolysis of coal under oxygen-limited conditions. Compared with biomass-derived biochar, coal-based charcoal generally exhibits a higher degree of carbonization and aromaticity, resulting in greater structural stability and longer persistence in soil [12,13]. These characteristics contribute to enhanced carbon sequestration potential and sustained improvements in soil physicochemical properties. However, the composition of coal-based charcoal is largely dependent on feedstock characteristics and production conditions, and it may contain trace amounts of potentially harmful elements, which could pose environmental risks under long-term use [14]. Therefore, it is essential to evaluate its agronomic performance and environmental suitability for the restoration of degraded soils.

Inner Mongolia is a major pastoral region with abundant livestock resources, providing substantial quantities of organic fertilizers, such as sheep manure [15]. Previous studies have demonstrated that combining carbon-based materials (e.g., lignite-derived products) with organic fertilizers can improve soil physicochemical and biological properties and enhance crop productivity [16,17]. Furthermore, the integrated utilization of local coal and livestock resources offers opportunities for local resource utilization and cross-sectoral development, linking agriculture, energy, and environmental management systems. This regional context offers a unique opportunity to explore integrated soil amendment strategies using locally available resources.

Existing research on soil amendment with coal-based charcoal and organic fertilizer has mainly focused on crops and herbaceous plants, including maize, wheat, rice, and various forage grasses [18,19]. These studies have demonstrated that such amendments can improve soil fertility, nutrient availability, and plant productivity. However, research on woody shrubs commonly used in ecological restoration, particularly drought- and salt-tolerant plants adapted to desertified environments, remains relatively limited [20,21,22]. *Amorpha fruticosa* L., a shrub species widely used for sand fixation and ecological restoration, exhibits strong adaptability to adverse conditions. However, the mechanisms underlying its morphological and physiological responses to combined soil amendments remain poorly understood. Therefore, the applicability of these soil amendments for restoring desert shrub ecosystems remains to be further elucidated.

These primarily include:

1. The mechanisms underlying the synergistic effects of coal-based charcoal and organic fertilizers, as well as their optimal application ratios, remain unclear. In particular, there has been a lack of systematic evaluation of their optimal application rates and the long-term stability of these materials in sandy soils with low organic matter content and high permeability.
2. Existing research has primarily focused on crops or general herbaceous plants as test subjects. Relatively little research has been conducted on the sand-fixation and soil restoration potential of the highly stress-tolerant shrub *A. fruticosa* L., and studies on its response mechanisms and adaptive strategies to integrated soil amendment measures remain insufficient, which has hindered the widespread application of related technologies in ecological restoration.
3. Most existing studies have focused on conventional indicators such as changes in soil physicochemical properties or plant productivity, and have not provided an in-depth analysis of the physiological and ecological mechanisms underlying seed germination, early growth, and enhanced stress tolerance. Consequently, a comprehensive framework linking soil improvement with plant response has yet to be established.

This study was conducted in Inner Mongolia, China. The effects of different proportions of coal-based charcoal and organic fertilizer on soil physicochemical properties, seed germination, and seedling growth of *A. fruticosa* L. were systematically evaluated. In the context of desertification control and given the region’s abundant coal resources, this study further explored the potential mechanisms by which the combined application of coal-based charcoal and organic fertilizer promotes the growth of *A. fruticosa* L., aiming to identify an optimal amendment strategy for restoring degraded soils.

## 2. Results

### 2.1. Effects of Coal-Based Charcoal and Organic Fertilizer on Soil Properties

**Figure 1:**
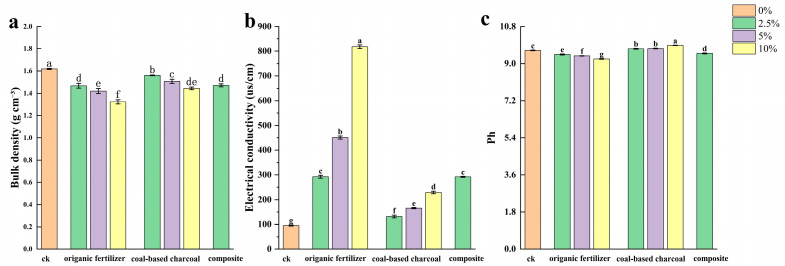
Bulk density (BD) (**a**), electrical conductivity (EC) (**b**), and pH (**c**) under different treatments. Different letters indicate statistically significant differences among treatments (*p* < 0.05).

As shown in Figure 1, different treatments significantly affected BD, electrical conductivity (EC), and pH. Compared with CK, all treatment groups exhibited varying degrees of reduction, ranging from approximately 9.3% to 20.77%. The reduction in bulk density may be attributed to the highly porous, low-density characteristics of coal-based charcoal and organic fertilizer, which increase soil pore space and improve soil structure. Additionally, organic fertilizer promotes the formation of soil aggregates by increasing organic matter content, thereby reducing particle packing and improving soil structure. Compared with CK, electrical conductivity (EC) increased significantly in all treatments (*p* < 0.05), with increases ranging from approximately 38% to 900%. Overall, EC showed an upward trend with increasing application rates, with the highest increases observed in the high-application-rate treatments (T10% and F10%). The FT treatment showed a 205% increase, indicating a certain degree of combined effect of coal-based charcoal and the organic fertilizer. These results indicate that the amended materials significantly increased soil soluble ion content, thereby enhancing nutrient availability. However, it should be noted that the increase in EC may pose a risk of salt stress, particularly at high application rates; their ecological effects still require further evaluation in conjunction with plant tolerance thresholds. In this study, although the EC value increased significantly, it remained below the plant tolerance threshold reported in the literature, indicating it is still within an acceptable range; however, long-term application still requires attention to salt accumulation [23,24].

There were significant differences in soil pH among the treatments (*p* < 0.05) (Figure 1c). The treatments involving sole application of organic fertilizers (F2.5%, F5%, F10%) significantly reduced soil pH, with a gradual decrease as the application rate increased; the F10% treatment had the lowest pH. In contrast, treatments with coal-based charcoal alone (T2.5%, T5%, T10%) significantly increased soil pH, with values rising as the application rate increased; the T10% treatment had the highest pH, significantly higher than both the CK and the organic fertilizer treatments. The soil pH in the FT treatment fell between organic fertilizer-only and high-dose coal-based charcoal treatments. However, FT did not reduce soil pH to the level of the CK treatment. Instead, its pH remained elevated and was comparable to that observed under the T2.5% treatment, indicating that the combined application only partially offset the alkalizing effect of coal-based charcoal.

### 2.2. Seed Germination Under Different Treatments

**Figure 2:**
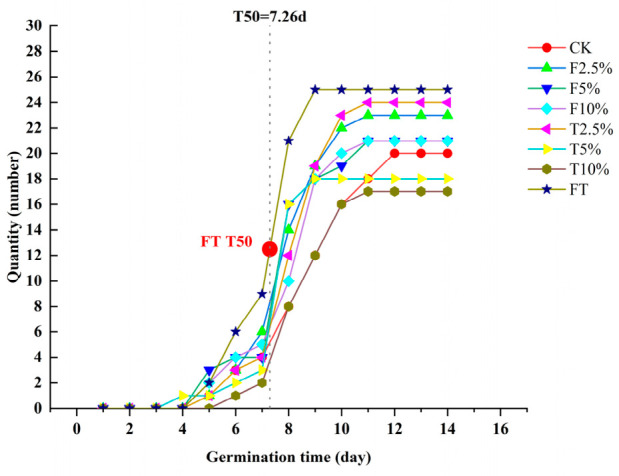
Germination dynamics of *A. fruticosa* L. under different treatments. The dashed vertical line and marker indicate the T_50_ value of the FT treatment (7.26 d).

Figure 2 illustrates the germination dynamics of *A. fruticosa* L. under different treatments over 14 days. Germination across all treatments occurred primarily between days 5 and 9, after which it gradually stabilized. At the early stage (Day 5), germination rates were generally low, although initial differences were observed among treatments; notably, the F5% treatment exhibited a relatively faster germination rate, while the coal-based charcoal treatments (T2.5%, T5%, and T10%) showed reduced early emergence. By day 7, all treatments entered a rapid germination phase, with FT showing the most pronounced increase, reaching a germination rate exceeding 25%, which was clearly higher than that of the other treatments. In contrast, higher coal-based charcoal application rates (T5% and T10%) exhibited relatively poor germination performance throughout this stage. After day 9, germination rates in most treatments slowed and approached a plateau. Final germination rates varied among treatments, with FT achieving the highest (83.33%), followed by T2.5% (80%) and F2.5% (76.67%), whereas T10% exhibited the lowest (56.67%). Overall, the results indicate that moderate combined application of coal-based charcoal and organic fertilizer promoted seed germination, whereas excessive coal-based charcoal application may inhibit it.

FT treatment significantly promoted the seed germination of *A. fruticosa* L., likely due to its multifaceted regulatory effects on the soil microenvironment. On the one hand, the porous structure of coal-based charcoal enhances soil water-holding capacity, reducing water loss in sandy soils and thus providing stable moisture conditions for seed germination. At the same time, the combination of these two amendments helps regulate soil pH and alleviates the inhibitory effect of salinity on seed germination, thereby promoting seedling emergence. In addition, from the perspective of germination kinetics, FT treatment resulted in a shorter time to reach 50% germination (T_50_) and a higher Germination Speed Index (GSI), indicating that it not only increased the final emergence rate but also accelerated seedling emergence. These results further suggest that the combined application of coal-based charcoal and organic fertilizer can significantly improve soil environmental conditions during seed germination, thereby promoting early seedling establishment.

As shown in Table 1, significant differences (*p* < 0.05) were observed among treatments in germination potential (GP), final germination rate (GR), germination index (Gi), and T_50_. The FT treatment exhibited the highest germination potential (30%) and final germination rate (83.33%), both of which were significantly higher than those in the CK treatment. The medium–low application treatments (F2.5% and T2.5%) also significantly improved germination compared with CK, whereas the high-rate coal-based charcoal treatment (T10%) exhibited the poorest germination performance. Significant differences were also observed in the germination index. T5% exhibited the highest Gi (54.17%), indicating a relatively rapid early germination response. However, its final germination rate remained lower than that of FT. This discrepancy reflects the distinct biological significance of these indices: Gi reflects early germination speed, whereas final germination rate reflects the overall germination success. The high Gi value for T5% suggests that germination occurred predominantly during the early stage, with subsequent germination limited. In contrast, FT exhibited the shortest T_50_ (7.26 d) together with the highest final germination rate. These results indicate that FT not only accelerated seed germination but also sustained germination over time, resulting in superior overall germination performance compared with the other treatments.

### 2.3. Effect of Coal-Based Charcoal and Organic Fertilizer Application on Root Morphology of A. fruticosa L.

**Figure 3:**
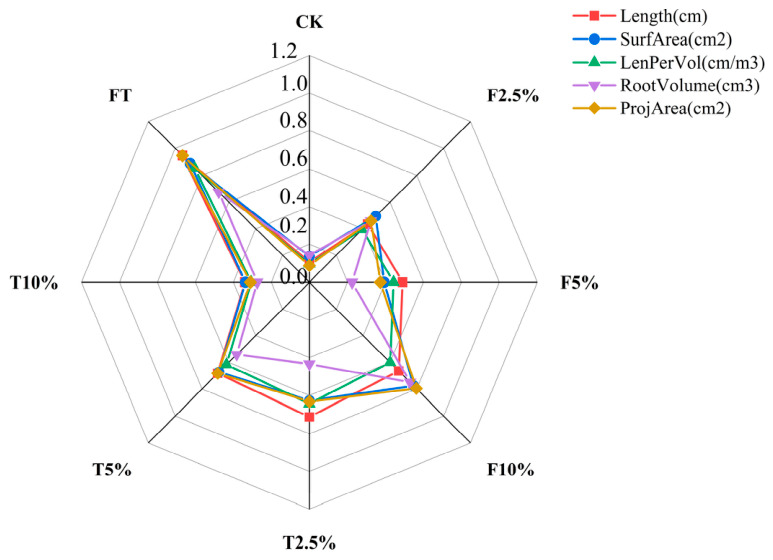
Effects of charcoal and organic fertilizer application on root morphological characteristics under different treatments.

As shown in Figure 3, soil amendment treatments significantly affected root morphological characteristics. Compared with the CK, all treatments increased root length, specific surface area, root density, root volume, and projected root area, although the magnitude of these effects varied among treatments. In the organic fertilizer treatments, root traits generally improved with increasing application rate, with F5% showing consistent improvements across multiple parameters and F10% achieving the highest values for most indicators. In contrast, the lowest application rate (F2.5%) showed relatively minor enhancement. For coal-based charcoal treatments, both T2.5% and T5% improved root development compared with CK, with T5% showing the strongest performance; however, further increasing the application rate to T10% led to a decline in most root parameters compared with the medium-rate treatment. The FT produced the most balanced and pronounced enhancement, with root length, root volume, and projected root area increased by approximately 30–80% compared with the CK treatment. The radar chart coverage area was significantly larger than for other treatments, and indicators such as root length, root-specific surface area, root volume, and projected root area remained at high levels, indicating better root morphological characteristics.

**Figure 4:**
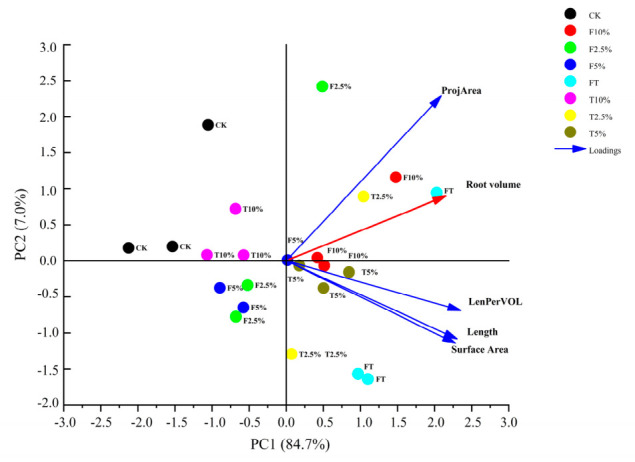
Biplot of principal component analysis of root morphological parameters of *A. fruticosa* L. under different treatments.

As shown in Figure 4, to comprehensively assess the effects of different treatments on the root morphology of *A. fruticosa* L., principal component analysis was used to analyze indicators including root length, root density, root specific surface area, root volume, and root projected area. The results showed that the first two principal components cumulatively explain 91.7% of the variance, with PC1 contributing 84.7% and representing the primary source of information. Regarding loading distribution, all root morphology indices showed positive loadings on PC1, indicating that PC1 primarily reflected overall root development. The positive correlations among all variables suggest that larger root systems tend to have greater root length, root density, specific surface area, root volume, and projected area. Root volume showed a relatively higher loading on PC2 than the other variables, indicating a greater contribution to the second principal component. However, this pattern mainly reflects its contribution to overall multivariate variation and should not be interpreted as evidence of a distinct treatment effect on root volume alone.

The different treatments tended to separate in principal component space. Treatments located in the positive region of PC1 generally exhibited higher values for the measured root morphological traits. Both FT and F10% were positively associated with PC1, indicating favorable root development. However, the overlap between these treatments in the biplot indicates that their overall root morphological profiles were largely similar in multivariate space. In contrast, treatments located in the negative region of PC1 generally had lower PC1 scores and weaker associations with the measured root traits.

### 2.4. Effects of Treatments on Plant Growth

#### 2.4.1. Effects on Plant Height

**Figure 5:**
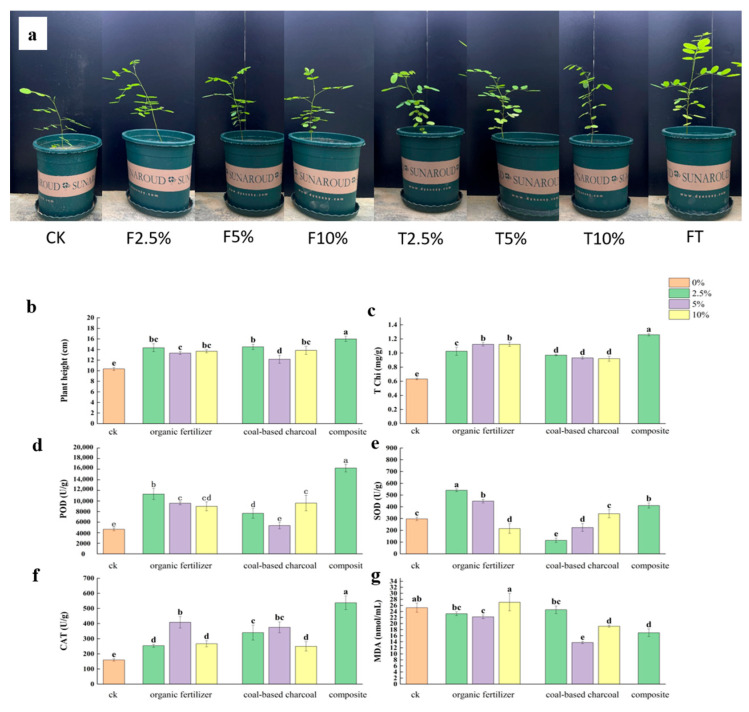
Effects of coal-based charcoal and organic fertilizer on plant height (**a**,**b**), chlorophyll content (**c**), POD activity (**d**), SOD activity (**e**), CAT activity (**f**), and MDA content (**g**) under different treatments. Different letters indicate statistically significant differences among treatments (*p* < 0.05).

Based on aboveground morphological characteristics, soil amendment treatments had different effects on plant growth (Figure 5a,b). Compared with the CK, all amendment treatments, including coal-based charcoal, organic fertilizer, and their combination, improved plant height, branching, and overall growth to varying degrees. Among these, FT exhibited the most pronounced growth, characterized by greater plant height, enhanced branching, and vigorous aboveground development, with fully expanded leaves and upright stems. These results indicate that the combined application significantly enhances aboveground growth compared with individual treatments.

#### 2.4.2. Effects on Physiological Characteristics

Different treatments significantly affected leaf chlorophyll content (Figure 5c). Compared with the CK, all treatments increased chlorophyll content to varying degrees, with organic fertilizer treatments generally performing better than charcoal treatments. In the coal-based charcoal treatments, chlorophyll content was higher at low application rates (T2.5%) but decreased at higher levels (T10%). The FT resulted in significantly higher chlorophyll content than the CK and most single treatments.

As shown in Figure 5d–g, antioxidant enzyme activities were significantly affected by the treatments. Compared with CK, all treatments increased the activities of peroxidase, superoxide dismutase, and catalase and reduced malondialdehyde content. The improvement was more pronounced at low to medium application rates, whereas higher application levels showed diminishing effects and, in some cases, slight reductions. Among all treatments, FT showed the most consistent and superior performance, with higher enzyme activities and lower oxidative stress levels than individual applications, indicating a more effective enhancement of the plant’s antioxidant defense system. The increase in antioxidant enzyme activity likely reflects a protective response, in which the improvement measures enhance the soil environment and promote moderate activation of the plant’s antioxidant defense system, thereby increasing its ability to scavenge reactive oxygen species and mitigating oxidative stress. In contrast, under high-application treatments, increases in some enzyme activities were attenuated or even reversed, suggesting that these changes may be related to secondary stress or nutrient imbalances induced by excessive application.

#### 2.4.3. Effects on Photosynthetic Characteristics

Different treatments significantly affected photosynthetic parameters (Table 2). Compared with CK, all amended treatments increased the net photosynthetic rate, transpiration rate, and stomatal conductance to varying degrees, although significant differences were observed among materials and application rates. Among all treatments, FT exhibited the highest photosynthetic rate, significantly exceeding those of CK and all single-application treatments. Among the organic fertilizer-only treatments, F10% showed the highest photosynthetic rate, significantly exceeding that of the CK and coal-based charcoal-only treatments. With increasing organic fertilizer application rates, transpiration rate and intercellular CO_2_ concentration generally increased. The concurrent changes in stomatal conductance, transpiration, and intercellular CO_2_ concentration suggest that stomatal behavior may have contributed to the observed photosynthetic responses, rather than these responses being solely attributable to increased CO_2_ assimilation capacity. In contrast, charcoal-only treatments moderately increased stomatal conductance and transpiration, but had a limited effect on photosynthetic rate. Moreover, WUE decreased with increasing coal-based charcoal application rates, suggesting that its effect on photosynthesis is mainly mediated by soil physical conditions and water regulation. The FT demonstrated superior performance, with higher photosynthetic rate and water-use efficiency, while maintaining relatively stable stomatal conductance and intercellular CO_2_ levels, suggesting synergistic effects of amendments.

### 2.5. Correlation Analysis of Soil Properties and Plant Growth

**Figure 6:**
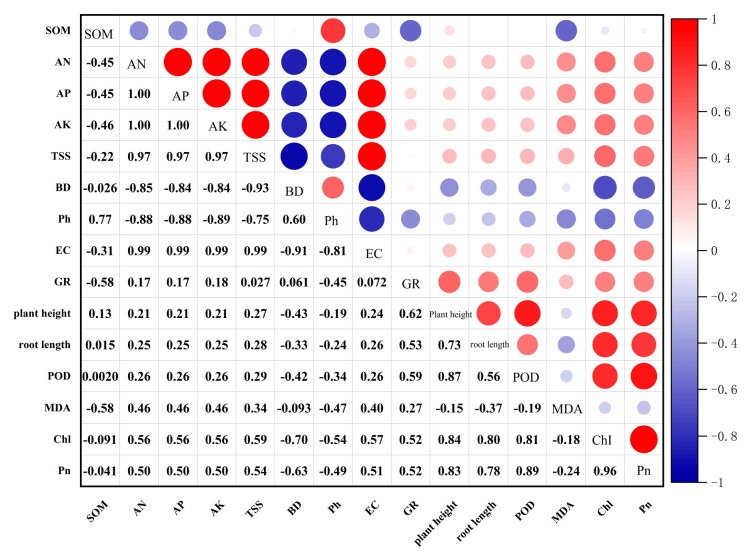
Pearson correlation analysis of soil properties, germination, and plant growth. The size of each circle is proportional to the absolute value of the correlation coefficient.

Correlation analysis revealed significant relationships among soil physicochemical properties, plant growth parameters, and physiological traits (Figure 6). Hydrolyzable nitrogen, available phosphorus, and available potassium showed strong positive correlations (r > 0.97) with total salt content and electrical conductivity, while exhibiting negative correlations with soil BD and pH, indicating that nutrient accumulation is associated with improved soil structure and changes in soil chemical properties. Organic matter displayed weak to moderate negative correlations with available nutrients, likely reflecting differences in the rates of organic matter decomposition and nutrient release.

In terms of plant growth, the 15-day germination rate showed moderate positive correlations (r = 0.52–0.62) with AN, AP, and AK, suggesting that early germination is sensitive to available nutrient supply. At 90 days, plant height was strongly positively correlated with root length (r = 0.73), and both were positively associated with available nutrients and negatively related to BD and pH, while showing only weak correlations with electrical conductivity. Regarding physiological traits, peroxidase activity showed significant positive correlations with plant height and root length (r = 0.56–0.87), whereas malondialdehyde content showed negative relationships with growth parameters, indicating reduced oxidative stress under favorable conditions. Chlorophyll content was strongly correlated with photosynthetic rate (r = 0.96) and also positively associated with plant height and root length, suggesting that enhanced photosynthetic capacity contributes to improved plant growth.

It should be noted that some soil nutrient indicators (such as HN, AP, and AK) exhibit high correlation coefficients (r > 0.97), indicating some degree of information overlap or multicollinearity among the variables. This phenomenon is relatively common in the context of synergistic changes in soil nutrients. Since the soil data in this study were analyzed using averages across treatments and the sample size was relatively small, this may have slightly inflated the degree of correlation among variables. Therefore, the above results are primarily intended to reveal trends in the relationships among variables and should not be used to infer strict causal relationships.

## 3. Discussion

### 3.1. Mechanisms Underlying Improvements in Soil Physicochemical Properties

The results demonstrate that the combined application of coal-based charcoal and organic fertilizer significantly improved the physicochemical properties of desertified soils. These improvements were reflected in reduced bulk density, increased porosity, increased soil pH, and increased soil organic matter and nutrient availability. These findings are consistent with previous studies showing that carbon-based amendments, owing to their large surface area, porous structure, and functional groups, enhance soil aggregation, increase porosity, and improve water retention and aeration, particularly in sandy and arid soils [25,26]. In parallel, organic fertilizer increases soil organic matter content and nutrient availability, thereby promoting soil fertility and plant growth [27,28].

From a mechanistic perspective, the contrasting chemical properties of the two amendments play a key role. Coal-based charcoal, which is generally alkaline, increases soil pH and enhances cation exchange capacity, thereby enhancing nutrient retention and reducing nutrient leaching losses. These findings are consistent with previous studies demonstrating that carbon-based amendments can increase soil pH, enhance nutrient retention, and improve soil chemical properties by interacting with the soil matrix through their surface functional groups [29,30].

In contrast, organic fertilizer tends to lower soil pH while supplying readily available nutrients. The interaction between these amendments creates a more balanced soil chemical environment, thereby enhancing nutrient availability and plant uptake. Similar synergistic effects have been reported for combined biochar–compost applications, which have been shown to improve soil structure, nutrient dynamics, and water retention [25,31,32].

Furthermore, the results indicate that single amendments primarily affect individual soil properties, whereas their combined application results in a more comprehensive improvement. Coal-based charcoal mainly affected soil pH and structural properties, while organic fertilizer contributed more substantially to nutrient enrichment and increased electrical conductivity. The FT, however, simultaneously improved soil structure, soil chemical balance, and nutrient status, highlighting the benefits of integrated amendment strategies for restoring desertified soils.

### 3.2. Effects on Growth and Physiological Responses and Underlying Mechanisms

The superior germination performance observed under the FT treatment may be attributed to improved soil water retention and a more balanced nutrient supply. High application rates of coal-based charcoal and organic fertilizer resulted in reduced performance and, in some cases, growth inhibition. These findings are consistent with previous studies showing that soil conditioners enhance plant productivity primarily by improving soil structure, moisture retention, and nutrient availability [33,34,35]. Enhanced root development suggests that the combined amendment created a more favorable rhizosphere environment. These changes facilitate deeper root penetration and lateral expansion in sandy soils, thereby improving nutrient uptake capacity [36,37].

Under FT treatment, the activities of superoxide dismutase (SOD), peroxidase (POD), and catalase (CAT) were significantly enhanced, while malondialdehyde (MDA) levels were markedly reduced, suggesting that the enhanced antioxidant defense system may be associated with improved cell membrane stability. SOD converts superoxide radicals (O_2_^−^·) into H_2_O_2_, which is subsequently detoxified by CAT and POD, thereby preventing lipid peroxidation [38,39]. The synergistic upregulation of CAT and POD activities observed in this study may indicate that the plant possesses high detoxification efficiency toward H_2_O_2_, thereby preventing its accumulation within cells and the induction of secondary oxidative stress. The reduction in MDA further reflects decreased membrane lipid peroxidation and improved stress resistance. Importantly, enhanced antioxidant activity was associated with improved plant growth rather than stress-induced inhibition, indicating that the treatment promotes a balanced physiological state conducive to growth [40]. Under these conditions, reactive oxygen species are less likely to accumulate as damaging agents and may instead function as signaling molecules that regulate plant growth processes [41].

Under the measurement conditions used in this study, soil amendments promoted chlorophyll synthesis and improved photosynthetic performance. Improved nutrient availability and soil moisture conditions may have contributed to the higher net photosynthetic rates and WUE. Notably, compared with F10%, the FT treatment achieved a higher Pn while maintaining relatively stable Ci. This pattern may reflect enhanced carbon assimilation; however, further measurements, such as chlorophyll fluorescence and carboxylation-related enzyme activity, are needed to verify this mechanism. Therefore, the improved photosynthetic performance under FT should not be attributed solely to increased stomatal conductance. This response is consistent with previous findings showing that nitrogen availability enhances CO_2_ carboxylation efficiency and mitigates the effects of environmental stress [42,43].

### 3.3. Dose–Response Effects and Potential Adverse Impacts

Moderate application rates of coal-based charcoal and organic fertilizer improved soil quality and plant performance, but higher rates (e.g., F10% and T10%) did not yield further improvements and, in some cases, led to declines in germination, growth, and photosynthetic parameters, suggesting the existence of an optimal application threshold beyond which additional inputs become counterproductive. Further analysis of the data from each treatment revealed that, at low to moderate application rates (such as T2.5%, F5%, and FT), growth and photosynthetic parameters generally performed well. However, when the application rate was increased to 10%, improvements in most indices slowed or even reversed, indicating that this system has an optimal application range. Based on comparisons among treatments, it can be concluded that the optimal application rate for coal-based charcoal and organic fertilizer is between 2.5% and 5%; within this range, their growth-promoting effects on *A. fruticosa* L. are relatively consistent. When the application rate exceeds this range, the growth-promoting effect tends to plateau or even become inhibitory. Excessive amendment application may substantially increase soil nutrient levels, pH, and electrical conductivity (EC), potentially reducing nutrient availability and inducing salt stress. These conditions may disrupt soil chemical balance and negatively affect plant physiological processes, thereby limiting growth performance. Similar patterns have been reported in previous studies, in which high carbon-based amendment inputs increased soil salinity and caused nutrient imbalances, pH shifts, and alterations in microbial interactions [44,45,46].

In addition, compared with CK, the F5% treatment significantly promoted plant growth, whereas the FT treatment exhibited greater overall benefits. For example, the germination rate under FT was 83.33%, higher than that under F5% (70%). Meanwhile, PCA and root radar chart analyses indicated that FT exerted a more balanced effect on root length, root volume, and overall root morphology. Moreover, FT significantly increased the activities of SOD, POD, and CAT while reducing MDA content, indicating improved stress resistance. The differences between F5% and FT suggest that the superior performance of FT cannot be explained solely by increased nutrient input. Although F5% supplied more nutrients, it was also associated with a greater increase in electrical conductivity (EC), which may increase the risk of osmotic stress in sandy soil environments. In contrast, the combined use of coal-based charcoal and organic fertilizer may create a more favorable soil environment. The porous structure of coal-based charcoal not only improved soil aeration and water-holding capacity but also enhanced nutrient retention and partially buffers soil pH fluctuations and salt accumulation. These findings further suggest that the combined treatment may help balance the benefits and potential risks associated with high application rates of individual amendments.

## 4. Materials and Methods

### 4.1. Soil Sampling

**Figure 7:**
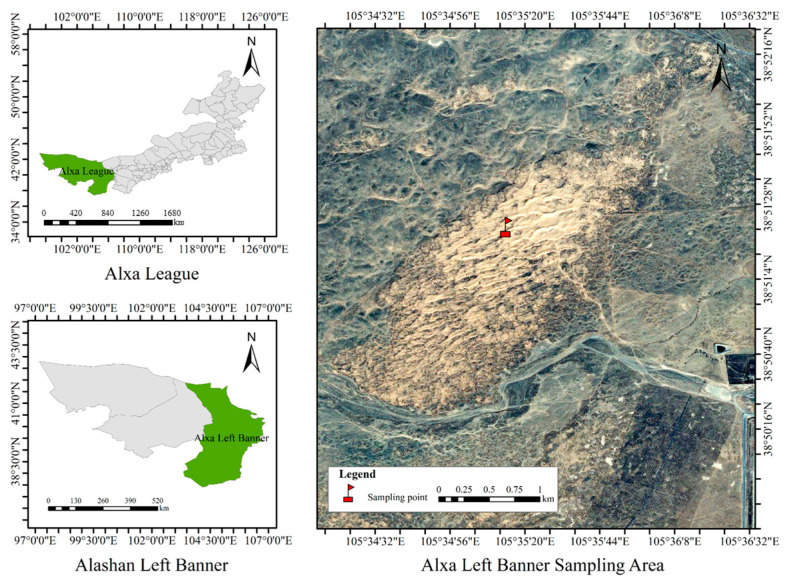
Location of the soil sampling sites in the Alxa region, Inner Mongolia, China. Note: The map is based on the official China Standard Map provided by the National Administration of Surveying, Mapping and Geoinformation (Review No. GS(2023)2767). The base map has not been modified.

Degraded sandy soil was collected from the western foothills of the Helan Mountains in the Alxa Left Banner region, Inner Mongolia Autonomous Region, China (38°50′32″–38°51′44″ N, 105°34′32″–105°35′44″ E). The sampling locations are shown in Figure 7. Topsoil samples (0–20 cm) were randomly collected, air-dried at room temperature, sieved through a 2 mm mesh, and homogenized prior to use.

Inner Mongolia is rich in coal resources; therefore, coal-based charcoal was selected as the carbon source for this study. The coal-based charcoal used in this study was obtained from coal pyrolysis residue produced by Xinjiang Guanghui Clean Refining & Chemical Co., Ltd. (Hami, Xinjiang, China). The charcoal was produced through low-temperature pyrolysis of coal under oxygen-limited conditions (approximately 500–600 °C). Before the experiment, the coal-based charcoal was air-dried, crushed, and sieved through a 2 mm sieve to ensure homogeneous mixing with the soil. Additionally, Inner Mongolia has abundant organic fertilizer resources, particularly livestock manure. The organic fertilizer used was mature, commercially available sheep manure supplied by Inner Mongolia Qingmu Agricultural Technology Co., Ltd. (Mongolia, China). The physicochemical properties and nutrient compositions of the coal-based charcoal, organic fertilizer, and soil are summarized in Table 3.

### 4.2. Experimental Design

**Figure 8:**
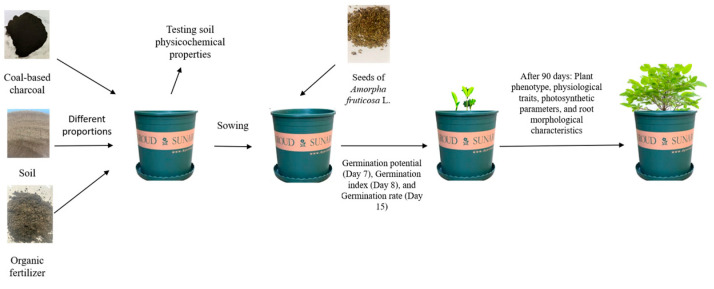
Schematic illustration of the potted plant experimental design.

Naturally air-dried soil was sieved through a 2 mm sieve, and then thoroughly mixed with coal-based charcoal and organic fertilizer according to the treatment design. As shown in Figure 8, the selection of application rates for coal-based charcoal was based on the ranges reported by [47], who used 2.5%, 5%, and 10% (*w*/*w*), and [48], who investigated 0.5%, 1%, 2%, 5%, and 10% (*w*/*w*). Based on previous studies, different application rates of coal-based charcoal and organic fertilizer were evaluated to assess their effects on soil properties and the growth performance of *A. fruticosa* L. Accordingly, eight treatments were established: CK (unamended desertified sandy soil), T2.5%, T5%, T10% (coal-based charcoal applied at 2.5%, 5%, and 10%, respectively), F2.5%, F5%, F10% (organic fertilizer applied at 2.5%, 5%, and 10%, respectively), and FT (2.5% coal-based charcoal + 2.5% organic fertilizer). In this study, the combined treatment (FT) consisted of 2.5% coal-based charcoal and 2.5% organic fertilizer, primarily based on the following considerations. On the one hand, this study focused on whether a low-dose combined application could achieve superior overall effects on sandy soil improvement and *A. fruticosa* L. growth compared with individual applications, without significantly increasing the risks of salt or nutrient imbalance. On the other hand, previous literature and this study’s results both suggest that higher application rates may increase electrical conductivity and pose a potential risk of salt accumulation. Therefore, this experiment primarily used a low-dose combined application scheme to preliminarily validate the potential for synergy.

To ensure sufficient space for the root growth of *A. fruticosa* L., plastic pots measuring 13 cm in height and 12 cm in diameter were used. Each pot was filled with 1 kg of soil, mixed with the appropriate amount of coal-based charcoal, organic fertilizer, or their combination, as specified in the treatment design. Ten seeds of *A. fruticosa* L. were sown in each pot. Each treatment included three replicates. To minimize interference from external environmental variables, all pots were randomly arranged in a controlled growth chamber at 25 °C, with a 16 h light/8 h dark photoperiod and 60–70% relative humidity. Uniform irrigation was applied throughout the cultivation period (50 mL of water was supplied every 3–4 days to each pot). The same irrigation regime was maintained across all treatments to ensure consistent water input and minimize the influence of irrigation variability on treatment comparisons.

### 4.3. Analytical Methods

#### 4.3.1. Soil Parameter Determination

Soil pH was determined using a pH meter (pH-3e, LEI-CI) in a 1:5 (*w*/*v*) soil–water suspension [49]. Electrical conductivity was measured using a conductivity meter (DDS-11A) in a 1:5 (*w*/*v*) soil–water extract [50]. Soil bulk density (BD) was determined as the mass of soil per unit volume of container. Soil organic matter (SOM) was measured using the potassium dichromate oxidation–titration procedure [51]. Soil cation exchange capacity (CEC) was determined using hexacyanoferrate extraction followed by spectrophotometric analysis [52].

The total nitrogen (TN) content of the soil was determined using the Kjeldahl method. The total phosphorus (TP) content was determined using the molybdenum–antimony colorimetric method. The total potassium (TK) content in the soil was measured using a flame photometer [53,54]. Available nitrogen (AN), phosphorus (AP), and potassium (AK) contents were determined using the alkaline hydrolysis diffusion method, sodium bicarbonate extraction–molybdenum–antimony colorimetric method, and ammonium acetate extraction–flame photometry method, respectively [55]. Total soluble salts (TSS) were determined following the method described by Lu [56]. Soil cations (Na^+^, Mg^2+^, Ca^2+^, and K^+^) were analyzed using an inductively coupled plasma emission spectrometer (ICP-OES; AVIO200) [57].

#### 4.3.2. Determination of Plant Indicators

(1) Germination parameters were recorded at different time points. Germination energy (Ge) was determined on Day 7, germination index (Gi) on Day 8, and germination rate (Gr) on Day 14. Germination rate (Gr) was calculated as (∑Gt/NT) × 100%, germination energy (Ge) as the number of seeds germinated within 7 days divided by NT, and germination index (Gi) as ∑(Gt/Dt), where Gt is the number of seeds germinated on day t, Dt is the corresponding germination time in days, and NT is the total number of seeds [58,59]. In addition, the time required to reach 50% germination (T_50_) was calculated according to the method of Coolbear et al. [60], as modified by Farooq et al. [61].

$${Τ}_{50}=t_{i}+\frac{\left(Ν÷2-n_{i}\right)}{\left(n_{j}-n_{i}\right)}\left(t_{j}-t_{i}\right)$$

where Ν is the final germination number, and $n_{i}$ and $n_{j}$ are the cumulative numbers of seeds germinated at adjacent times $t_{i}$ and $t_{j}$, respectively, when $n_{i}<Ν/2<n_{j}$.

(2) Root samples were carefully excavated at harvest and gently washed with tap water to remove adhering soil particles. The roots were stored at −80 °C and then thawed at room temperature before scanning. Root samples were evenly spread in a transparent tray containing a thin layer of water to minimize overlap, and the tray was scanned using an EPSON Perfection V850 Photo scanner (Seiko Epson Corporation, Suwa, Japan) at 600 dpi. The scanned images were analyzed using WinRHIZO Pro 2007 (Regent Instruments Inc., Quebec City, QC, Canada) with automatic grayscale thresholding and identical parameter settings for all samples. Root length, projected area, surface area, average diameter, and root volume were obtained from the software outputs. Principal component analysis (PCA) was used to reduce the dimensionality of variables such as root length, root specific surface area, root density, root volume, and projected root area, and to comprehensively evaluate the effects of different treatments on root morphological traits [62,63]. The analysis was conducted using Origin 2021b software, and the data were standardized prior to analysis to eliminate the influence of measurement differences.

(3) Plant morphology was recorded by photographing plants under different treatments at 90 days after planting.

(4) Seedling height was measured at 90 days after sowing using a ruler.

(5) On the 90th day after sowing, the third to fifth fully expanded leaves from the apex were selected for photosynthetic measurements. One representative plant with uniform growth was randomly selected from each of the three replicate pots for each treatment, and three fully expanded leaves were selected per plant. Photosynthetic parameters, including net photosynthetic rate (Pn), transpiration rate (Tr), stomatal conductance (Gs), intercellular CO_2_ concentration (Ci), and water use efficiency (WUE), were determined using an HM-GH40 portable photosynthesis system. Measurements were conducted between 09:00 and 11:30 h [64]. For each leaf, the instrument automatically recorded five consecutive readings, and the mean value was used for subsequent analyses. All measurements were performed under identical chamber conditions, with a leaf area of 2 cm^2^, atmospheric pressure of approximately 1013.2 kPa, chamber temperature of 32.2–34.2 °C, leaf temperature of 27.8–29.9 °C, and photosynthetic photon flux density of approximately 100–105 μmol/m^2^ s.

(6) Physiological parameters were determined as follows: Harvest seedlings grown for 90 days, take 0.1 g of leaf samples, wrap the samples in aluminum foil, and immediately immerse them in liquid nitrogen for freezing, and then store them at −80 °C in a freezer for subsequent testing of chlorophyll, SOD, POD, CAT, and MDA. The chlorophyll content was measured using a UV spectrophotometer after 48 h of soaking, as described by Dudek et al. [65]. Detection of SOD, POD, CAT, and MDA: Follow the kit instructions carefully. Take 0.1 g of frozen leaves, place them in a mortar, add 1 mL of the appropriate extraction solution, and homogenize on ice. Then centrifuge at 8000× *g* for 10 min at 4 °C, collect the supernatant, and keep it on ice for later use. When measuring different contents, appropriate dilutions are required, and the corresponding dilution factor should be applied in the final calculation. The SOD Activity Assay Kit (BC5165), POD Activity Assay Kit (BC0090), CAT Activity Assay Kit (BC0205), and MDA Content Assay Kit (BC6415) were purchased from Beijing Solarbio Science & Technology Co., Ltd. (Beijing, China)

### 4.4. Data Analysis

Data were organized and analyzed in Microsoft Excel 2019. All data are presented as ‘Mean ± Standard Deviation’ (Mean ± SD). Before performing analysis of variance, the Shapiro–Wilk test was used to assess normality, and the Levene test was used to assess homogeneity of variance. If both normality and homogeneity of variance are satisfied, a one-way analysis of variance is performed. Box plots were used to identify potential outliers. If an observation deviates from the mean by more than 3 standard deviations, it is considered an outlier and removed. After removal, normality and homogeneity of variance tests are repeated. Differences among treatments in morphological and physiological parameters were determined using Duncan’s multiple range test at a significance level of α = 0.05. Pearson’s correlation analysis was conducted to evaluate the relationships among growth traits, physiological parameters, and soil properties of *A. fruticosa* L. Graphs were generated using Origin 2021b.

## 5. Conclusions

This study demonstrates that the combined application of coal-based charcoal and organic fertilizer is an effective strategy for improving soil properties and enhancing plant performance in desertified areas. The results showed that soil amendments reduced bulk density, enhanced nutrient availability, and regulated soil chemical properties, with coal-based charcoal primarily increasing pH and organic fertilizer contributing to nutrient enrichment. The combined application provided a more balanced soil environment by mitigating excessive pH shifts and simultaneously improving soil structure and fertility.

Among the tested treatments, the combined application of FT at a moderate rate consistently showed the best performance, significantly enhancing seed germination, root development, and overall plant growth. This improvement was associated with enhanced root system architecture, facilitating more efficient water and nutrient acquisition in sandy soils. In addition, FT improved physiological performance by increasing chlorophyll content, enhancing photosynthetic performance, and strengthening the antioxidant defense systems, as reflected by increased SOD, POD, and CAT activities and reduced MDA levels. These responses indicate enhanced stress regulation and a more favorable physiological state, both of which support sustained growth.

Correlation analysis further revealed that plant growth was strongly associated with soil nutrient availability, photosynthetic performance, and antioxidant activity, highlighting the coupled roles of soil improvement and physiological regulation in driving plant growth. However, excessive application of either amendment did not provide additional benefits and, in some cases, adversely affected plant growth, indicating a clear dose–response relationship and the importance of maintaining optimal application rates. Overall, this study highlights that a moderate combined application of coal-based charcoal and organic fertilizer offers a practical and effective approach for restoring desertified soils, improving soil quality, and enhancing plant physiological performance. These findings provide a scientific basis for optimizing soil amendment practices in arid and semi-arid regions.

## 6. Limitations and Future Perspectives

Despite the positive effects of combining coal-based charcoal with organic fertilizer on soil improvement and plant growth observed in this study, several limitations should be acknowledged. First, the experiment was conducted under controlled pot conditions and therefore may not fully represent the complex environmental conditions of desertified field ecosystems. Factors such as rainfall variability, wind erosion, diurnal temperature fluctuations, and soil heterogeneity may influence the effectiveness of soil amendments under field conditions. In addition, although three biological replicates were established for each treatment, the relatively limited number of replicates may limit the statistical power to detect subtle differences among treatments. While this level of replication is commonly accepted in exploratory pot experiments, caution is still required when extrapolating the results to large-scale ecological restoration practices. Therefore, future studies should incorporate larger sample sizes and field-scale experiments to further validate the robustness and practical applicability of the findings. Second, this study evaluated plant and soil responses only over 90 days. Although no obvious adverse effects associated with increased electrical conductivity (EC) were observed during the experiment, it remains unclear whether long-term, repeated applications may lead to salt accumulation, thereby affecting soil sustainability. Therefore, long-term field experiments are needed to further assess the ecological safety and sustainability of these amendment strategies.

Third, only one combined application ratio (2.5% coal-based charcoal + 2.5% organic fertilizer) was evaluated in this study. Although this treatment exhibited the best overall performance, the optimal application ratio and its stability under different environmental conditions remain uncertain. Finally, a biomass-derived biochar treatment was not included as a control. Therefore, a direct comparison between coal-based charcoal and conventional biochar was not possible. Although the results demonstrated that the combined application of coal-based charcoal and organic fertilizer significantly improved soil properties and plant growth, it remains unclear whether these benefits are specific to coal-based charcoal. Future studies should incorporate biomass-derived biochar under identical experimental conditions, compare multiple combined application ratios, and conduct long-term field trials to further elucidate the underlying mechanisms and evaluate their practical applicability for restoring degraded soils.

## Figures and Tables

**Table 1:** Germination potential, germination rate, germination index, and T_50_ of *A. fruticosa* L. under different treatments (mean ± SD).

Treatment	Ge (%)	Gr (%)	Gi (%)	T_50_ (d)
CK	13.33 ± 5.77 bc	66.67 ± 5.77 abc	16.67 ± 7.22 c	8.58 ± 0.38 a
F2.5%	20 ± 10 ab	76.67 ± 11.54 ab	33.33 ± 14.43 bc	7.63 ± 0.33 bcd
F5%	13.33 ± 5.77 bc	70 ± 10 abc	50 ± 12.5 ab	7.53 ± 0.21 bcd
F10%	16.67 ± 5.77 bc	70 ± 10 abc	20.83 ± 7.22 c	8.06 ± 0.1 abc
T2.5%	13.33 ± 5.77 bc	80 ± 10 a	33.33 ± 7.22 bc	7.97 ± 0.21 abc
T5%	10 ± 0 bc	60 ± 10 bc	54.17 ± 7.22 a	7.47 ± 0.14 cd
T10%	6.67 ± 5.77 c	56.67 ± 5.77 c	25 ± 12.5 c	8.17 ± 0.76 ab
FT	30 ± 10 a	83.33 ± 5.77 a	50 ± 12.5 ab	7.26 ± 0.23 d

Note: Values within the same column followed by different lowercase letters indicate significant differences among treatments at *p* < 0.05. Values are presented as mean ± SD.

**Table 2:** Photosynthetic characteristics under different treatments (mean ± SD).

Treatment	Pn	Tr	Gs	Ci	WUE
CK	0.50 ± 0.05 e	0.06 ± 0.008 f	0.0059 ± 0.0002 c	532.1 ± 17.64 e	5.4601 ± 0.95 bc
F2.5%	1.04 ± 0.03 c	0.12 ± 0.004 d	0.0049 ± 0.0001 e	567.53 ± 7.3 bc	6.2090 ± 0.05 ab
F5%	1.26 ± 0.08 b	0.14 ± 0.01 c	0.0051 ± 0.0002 e	574.86 ± 9.84 b	7.6207 ± 0.7 a
F10%	1.29 ± 0.07 b	0.16 ± 0.001 b	0.0056 ± 0.0002 d	592.6 ± 8.43 a	6.5717 ± 1.03 ab
T2.5%	0.94 ± 0.1 cd	0.14 ± 0.006 c	0.0067 ± 0.0001 a	549.79 ± 1.03 d	4.2243 ± 1 cd
T5%	0.9 ± 0.01 d	0.1 ± 0.009 e	0.0065 ± 0.00008 ab	553.87 ± 7.39 cd	3.7332 ± 1.3 cd
T10%	0.99 ± 0.07 cd	0.11 ± 0.014 de	0.0064 ± 0.00006 b	547.71 ± 4.79 d	2.7929 ± 1.34 d
FT	1.74 ± 0.1 a	0.20 ± 0.006 a	0.0067 ± 0.0002 a	557.46 ± 4.88 cd	7.1993 ± 0.97 ab

Note: Values within the same column followed by different lowercase letters indicate significant differences among treatments (*p* < 0.05). Data are presented as mean ± SD. Measurement conditions for photosynthetic parameters, including light intensity, temperature, and CO_2_ concentration, are described in Section 4.3.2.

**Table 3:** Properties of coal-based charcoal, organic fertilizer, and soil.

Properties	Content		
	Coal-Based Charcoal	Organic Fertilizer	Soil
pH	9.8	7.59	9.22
TSS (g/kg)	1.79	35.5	0.67
CEC (cmol/kg)	1.94	29.1	1.34
SOM (g/kg)	509	26.2	2.13
TN (g/kg)	9.582	11.25	0.17
TP (g/kg)	0.0276	3.14	0.31
TK (g/kg)	2.13	26.2	18.3
AN (mg/kg)	8	29.1	8
AP (mg/kg)	4.7	290.4	14.3
AK (mg/kg)	10	16,129	59
Water-soluble potassium (g/kg)	0.00149	6.75	0.0216
Water-soluble calcium (g/kg)	0.115	0.483	0.0664
Water-soluble sodium (g/kg)	0.361	3.56	0.0409
Water-soluble magnesium (g/kg)	0.00645	0.418	0.0192

Note: The above data was tested according to the forest soil method index measurement method. For detailed methods, see Section 4.3.1 Soil Indicator Measurement. TSS, total soluble salts; CEC, cation exchange capacity; SOM, soil organic matter; TN, total nitrogen; TP, total phosphorus; TK, total potassium; AN, available nitrogen; AP, available phosphorus; AK, available potassium.

## Data Availability

The data presented in this study are available within the article. Additional data are available from the corresponding author upon reasonable request.
